# The impact of vehicle moving violations and freeway traffic flow on crash risk: An application of plugin development for microsimulation

**DOI:** 10.1371/journal.pone.0184564

**Published:** 2017-09-08

**Authors:** Junhua Wang, Yumeng Kong, Ting Fu, Joshua Stipancic

**Affiliations:** 1 College of Transportation Engineering, Tongji University, Shanghai, China; 2 Department of Civil Engineering and Applied Mechanics, McGill University, Montreal, Canada; Chongqing University, CHINA

## Abstract

This paper presents the use of the Aimsun microsimulation program to simulate vehicle violating behaviors and observe their impact on road traffic crash risk. Plugins for violations of speeding, slow driving, and abrupt stopping were developed using Aimsun’s API and SDK module. A safety analysis plugin for investigating probability of rear-end collisions was developed, and a method for analyzing collision risk is proposed. A Fuzzy C-mean Clustering algorithm was developed to identify high risk states in different road segments over time. Results of a simulation experiment based on the G15 Expressway in Shanghai showed that abrupt stopping had the greatest impact on increasing collision risk, and the impact of violations increased with traffic volume. The methodology allows for the evaluation and monitoring of risks, alerting of road hazards, and identification of hotspots, and could be applied to the operations of existing facilities or planning of future ones.

## Introduction

Vehicle moving violations are a major cause of traffic crashes and road fatalities, contributing to 75% of road crashes in China [1], 50% of all fatal crashes in Europe [2], and 56% of fatal crashes in the United States [3]. Rear-end collisions are the most common type of freeway crashes [4], representing 34% of all road crashes in China [5] and 51% of crashes on an American highway [6]. On freeways, vehicle violations reduce headways and reaction times creating more crashes while high travel speeds generate more severe crashes [7]. Speeding, slow driving, and abrupt stopping are behaviors closely related to rear-end collisions [5] [8] and evaluating their impact on freeway safety is essential in reducing crashes and preventing loss of life. Of the studies using historical crash data to evaluate the impact of violations on safety [1] [9] [10], very few have considered the actual mechanism between violations and crash risk.

The impact of violating behaviors is both microscopic and macroscopic. Microscopically, unexpected maneuvers from violating vehicles have a direct impact on surrounding vehicles (increased likelihood of conflicts). Macroscopically, violating vehicles indirectly impact traffic flow by reducing average speeds and headways (increased turbulence in traffic). Predicting crash risk and estimating the impacts of vehicle violations on freeway safety would assist in improving road safety countermeasures, operations and safety management of existing roads, and planning of future facilities. However, other than including vehicle violations as a factor in traditional crash models, not much has been explored. While capturing violating behaviors in real traffic is difficult and dangerous, traffic simulation produces virtual road scenarios that closely mimic reality and has been widely used in research and practice to design, operate, and evaluate transportation systems [11]. Simulated safety analysis does not require real collisions to occur, allowing for the investigating of road safety in a microscopic way, at the individual behavior level, and a macroscopic way, exploring the influence on traffic overall.

The purpose of this research is to develop and present algorithms for including vehicle violations in traffic simulation, to present a collision probability algorithm, to propose a framework for estimating the macroscopic impacts of violations, and to demonstrate this framework in a simulation experiment. This paper provides several contributions to the existing state of research. First, most studies simulating the impact of traffic risks have assumed ideal vehicle behavior [12] [13] [14]. This study presents a method for simulating various driver violating behaviours in existing microsimulation software through user-defined add-ons. Second, this paper adds to the state-of-the-art research on freeway safety assessment by improving methods for simulated safety analysis. Though time-to-collision of stopping-sight-distance are some of the most promising surrogate measures for assessing freeway safety, the lack of violating behaviours in existing software has made these measures difficult to evaluate in simulated environments. Third, by simulating individual violating behaviours, it is possible to examine their microscopic and macroscopic impact on freeway safety. Though some studies have explored the microscopic impact of vehicle maneuvers and violations, no studies have considered the macroscopic impact of violating behaviors. This paper proposes a method for analyzing collision risk of various violating behaviours.

## Literature review

The highlights of this work are directly related to the main areas of interest reviewed in the following sections, namely vehicle behavior, surrogate safety measures, and freeway collision risk.

### Traffic simulation and vehicle behavior

Traffic simulation techniques have improved since the late 1950s, from simple simulation for studying signal control [15] to advanced simulations for traffic planning, road design, safety evaluation, and behavioral analysis. Traffic simulation, as defined by the FHWA, includes three levels. Macroscopic simulation focuses on relationships between flow, speed, and density of the traffic stream. Microscopic simulation simulates the movement of individual vehicles using car-following and lane-changing models. Mesoscopic simulation falls between micro- and macroscopic approaches [16]. Numerous traffic simulation programs have been developed, including common packages such as Aimsun, SimTraffic, VISSIM, and EMME [16] [17]. As violations relate to vehicle behaviors, simulating vehicle violations and observing their impact on surrounding vehicles can be realized through microscopic simulation. The impact of violating behaviors on traffic flow is one promising application of mesoscopic simulation, requiring a microscopic software platform capable of reproducing precise vehicle violations and simultaneously describing macroscopic traffic scenarios. Simulation is likely “the only way to test driver/traffic models for safety applications” [18], and is a good solution for investigating the impact of driving violations on freeway safety. Studies to date largely focus on aggressive driving behaviors rather than explicitly considering violations and their macroscopic impacts. Li compared three simulation platforms for investigating aggressive lane-changing behaviors [19]. Punzo & Ciuffo utilized the SCANeR program in combination with Aimsun to investigate non-normative driving behavior [18]. Habtemichael & de Picado Santos used VISSIM to explore the impact of aggressive driving on collision likelihood and severity under congested and uncongested conditions and found that impact of aggressive driving increases in congestion [20]. This work contributes to the literature with developed methods for simulating various violating behaviours.

### Traffic simulation and surrogate safety measures

Studies investigating road safety using traffic simulation rely on surrogate safety measures (SSMs), which have gained popularity in road safety analysis [21]. Though many different measures have been proposed [22] [23], the most popular SSMs for simulation are summarized by Gettman and Head [24] and include time-to-collision (TTC) [25], stopping distance index [26] [27], modified time-to-collision [28], and vehicle speeds and headways [29]. Piao and McDonald studied the impact of speed limits on safety in different motorway sections using SSMs including headway, speed difference, and frequency of lane changes in Aimsun [30]. Habtemichael et al. used VISSIM and the Surrogate Safety Assessment Model (SSAM) [31] to analyze the crash risk, severity level, and magnitude of perceived benefits (e.g. time saved) of aggressive driving under congested and non-congested conditions [32]. Work on this topic remains limited and insufficient.

The most promising measures for investigating freeway safety include TTC and stopping sight distance (SSD). Both measures represent the available time and distance for a driver to attempt evasive action, which is closely related to freeway crashes. TTC is the time remaining before two vehicles collide if they maintain their current speed and direction, and is correlated with rear-end collision occurrence [33]. Several studies have used TTC to estimate freeway collision risk [30] [34] [35]. However, TTC techniques do not consider varying driver response characteristics, are incapable of estimating risks during unexpected events including violating behaviours, do not reflect conflict severity, and require the following vehicle to travel faster than the leading one in order to estimate rear-end collision risk. For these reasons, TTC may not work well for erroneous or risky driver behavior [36] and analyzing vehicle violations.

**Fig 1:**
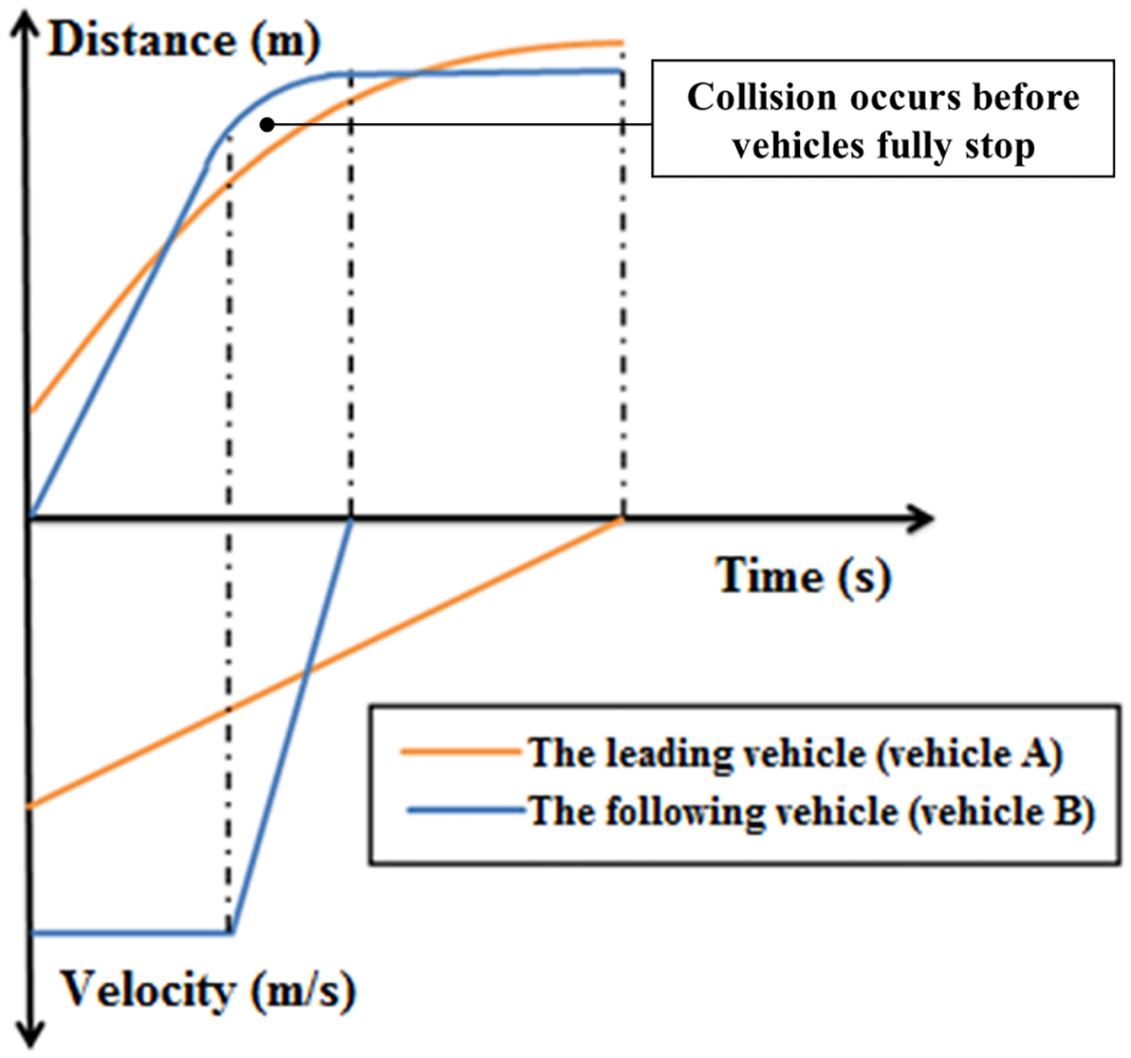
Vehicle collision distance-time-velocity diagram.

SSD describes conflicts in a more microscopic way and considers different confounding factors. SSD is the sum of braking reaction distance (distance travelled during perception-reaction time) and braking distance (distance travelled during deceleration) [37] [38]. Though SSD has been used effectively to measure rear-end conflicts in freeways, the measure is limited in situations where vehicle pairs have a significant speed difference. Assume that leading vehicle *A* and following vehicle *B* have an initial distance gap of ***L***. At *t = 0*, *A* brakes suddenly, followed by *B* after a perception-reaction period. The vehicle trajectories are illustrated in the space-time diagram in Fig 1. Set the braking distance of *A* as ***D***_***A***_ (for *B*, ***D***_***B***_). By the SSD approach, situations are safe when ***D***_***B***_ < ***D***_***A***_ + ***L***. This measure is not reliable when the leading and following vehicles have significantly different speeds and deceleration rates, as vehicles may collide before stopping completely even if ***D***_***B***_ < ***D***_***A***_ + ***L***. Clearly, a more accurate model is needed. Though SSMs in general appear to be feasible [39] [40], they require additional improvement before their widespread acceptance. By incorporating violating behaviors in microsimulation, this paper enables simulated surrogate safety analysis of freeway facilities.

### Traffic simulation and freeway collision risk

Macroscopic traffic flow and freeway safety have been investigated using two approaches [41, 42]. In the aggregate approach, the safety performance of a fixed area is considered and impacts of “mobility characteristics, the transport system and the socio-economic characteristics of the area itself” are uncovered [42]. The disaggregate approach investigates collision risks at the event level, such as estimating collision probability by analyzing driver behavior [42]. The disaggregate approach should be applied when considering the impact of vehicle violations. Disaggregate studies of freeway safety typically rely on traffic conflict techniques, including conflicts identified using TTC thresholds. Zhou et al. investigated collision risks in the freeway network around Ningbo, China based on conflict counts and traffic volumes [43]. Jiang et al. used counts of dangerous conflicts as the criteria for evaluating collision risks over a freeway network in real-time [44]. Some studies have considered the distribution of SMM values (commonly TTC) and used their quintiles as thresholds for determining high-risk states. For example, Xiang et al. proposed the 85^th^ percentile of TTC values as the collision risk threshold to identify high risk states in freeway segments [45].

Simulation techniques allow for evaluation and prediction of safety in large road networks, which is difficult using field-observed data. Song & Sun used VISSIM to identify expressway on-ramp bottlenecks for safety and operational analysis purposes [46]. Yan et al. investigated the causes of crashes at freeway ramps using VISSIM [47] by creating a collision risk index based on the deceleration rate required for a following vehicle if the leading vehicle stopped abruptly. Young et al. summarized past work on measuring road safety using simulation [48]. Freeway safety requires more consideration and more advanced safety, while violating behaviors, as key factors contributing to crashes, should be included in simulation. The final contribution of this paper is presenting a method for evaluating the safety of freeway road sections.

## Methodology

**Fig 2:**
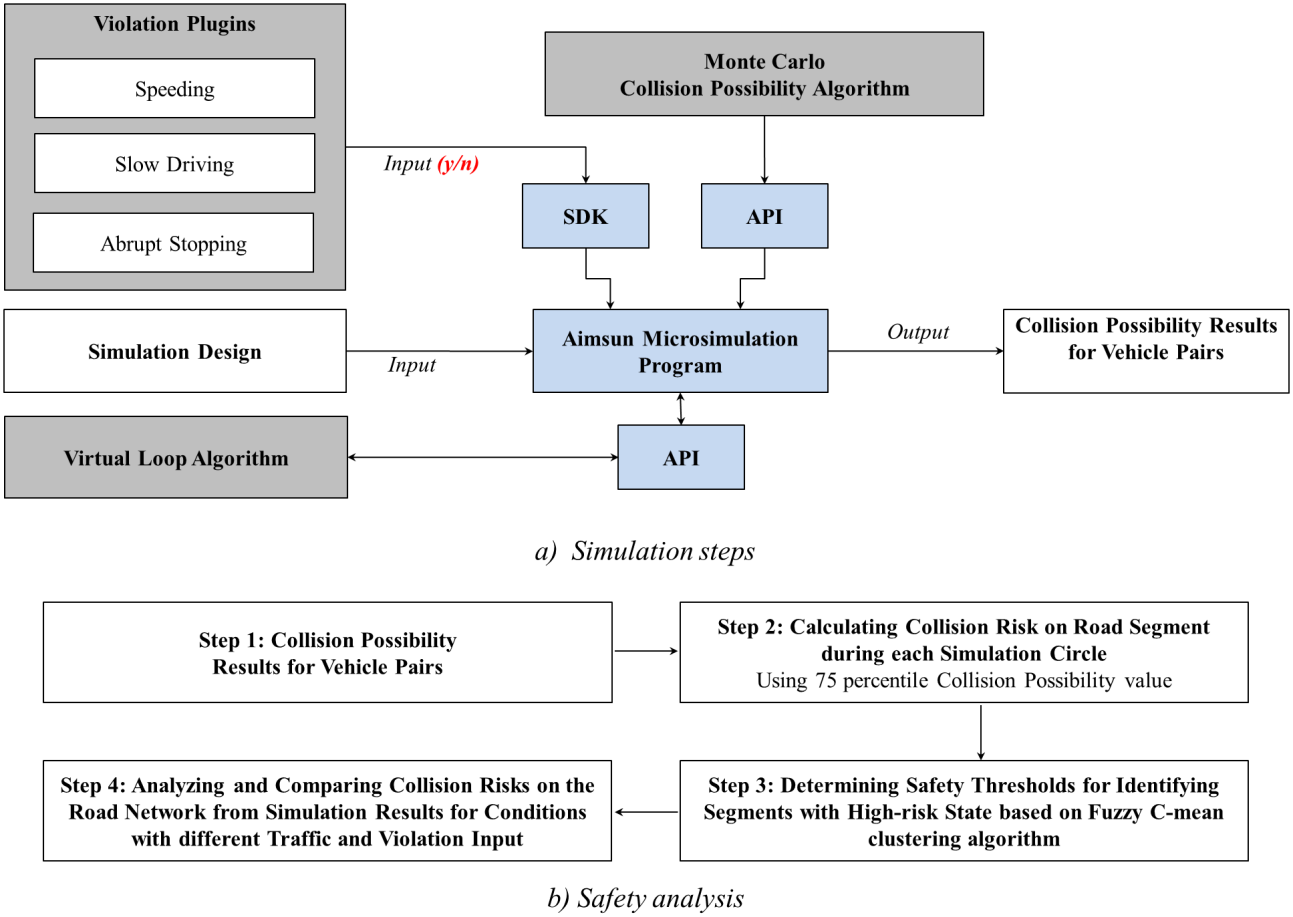
Methodology framework.

The methodology for this project consists of four steps. First, the Aimsun microsimulation program, along with its API and SDK modules, are introduced. Second, the plugins for programming and simulating vehicle moving violations are presented. Third, a Monte-Carlo model for estimating collision probability between vehicle pairs on freeway segments is proposed. Fourth, a Fuzzy C-mean clustering algorithm is used to identify high-risk freeway segments based on the risk probability data for vehicle pairs. In total, four applications were developed which contribute to the existing state of research. Three algorithms for simulating moving violations and the Monte-Carlo collision probability model were created. Fig 2 illustrates the entire methodology.

### Aimsun microsimulation program

The Aimsun program, developed by Transport Simulation Systems (TSS), was selected for this project because it is widely used, is efficient for simulating traffic and vehicle behavior, provides a user-friendly software interface, and is semi-open source software which allows for secondary development of simulation models, including vehicle violations [49]. Aimsun uses the widely accepted Gipps’ vehicle-following and lane changing models, which are effective at describing the behavior of interacting vehicles [50]. The vehicle-following model considers two traits of the following vehicle; intention to drive at an expected speed and actual speed of the following vehicle attempting to reach the expected speed. Gipps’ model considers human factors of driver expectation and reaction and accurately describes vehicle-following behavior in non-congested situations [51]. The model can be calibrated for non-steady-state conditions related to aggressive driving or violations [51]. The model for this study was calibrated on real-road traffic data collected from the Zhajiasu Expressway [52].

Another key driver behavior model for simulation is the lane change model. Aimsun uses a further development of Gipps’ lane change model [53], where the decision to change lanes is determined by three factors: 1) whether the driver can drive at their expected speed; 2) whether it is desirable to change lanes, and; 3) whether it is possible to change lanes [54]. The lane change model precisely mimics the entire lane change procedure from the driver perspective, including perception, judgement, and action, with consideration of traffic flow factors of speed and position and roadway geometric design [55]. These models can be manipulated to simulate aggressive driving or violations.

#### API module

**Fig 3:**
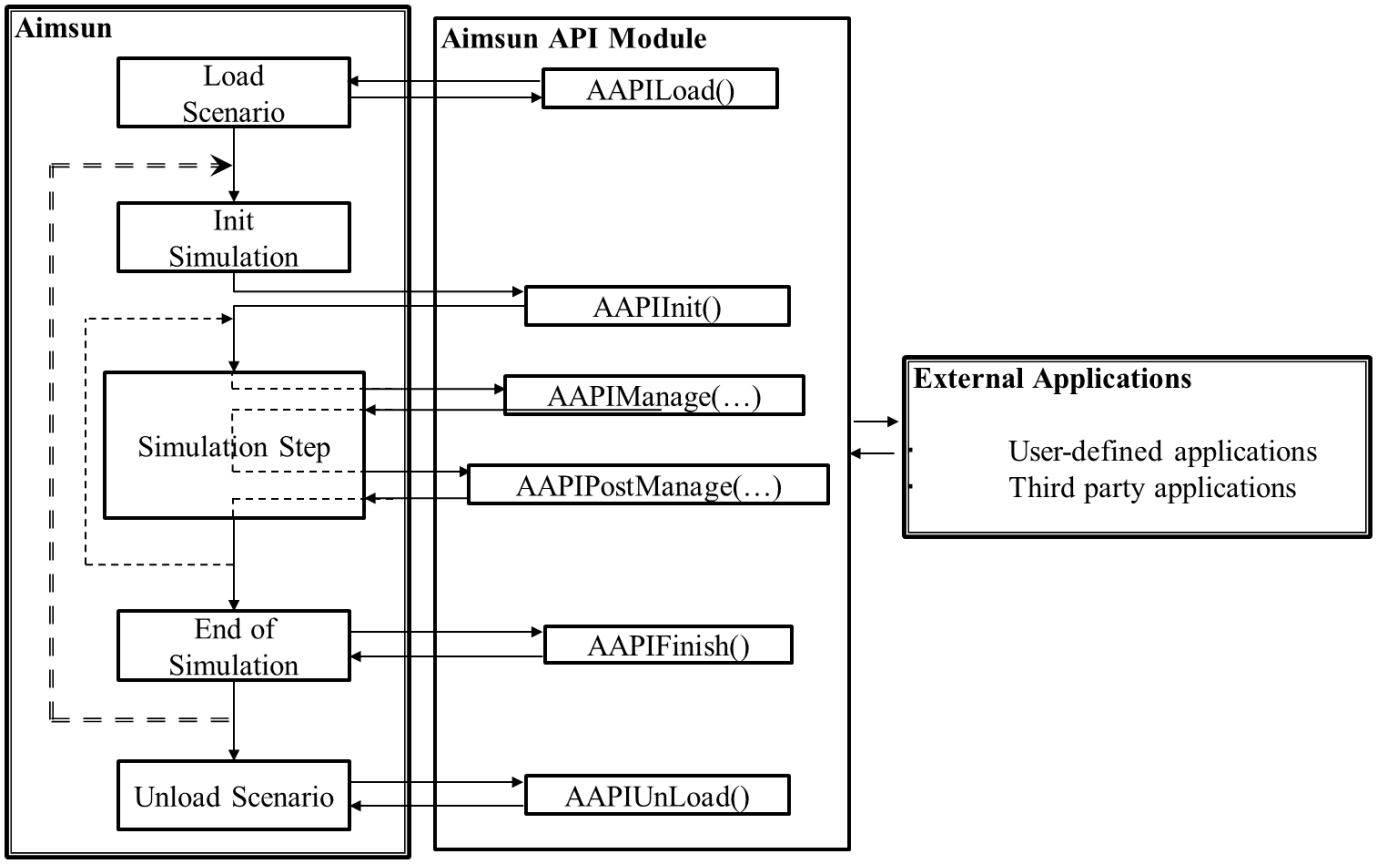
API module application flowchart.

The Application Programming Interface (API) enables users to implement an interface connection to user-defined and third party applications [56] [57] to improve the default simulation settings. Fig 3 presents the flowchart of the Aimsun API. The API module contains six main functions for communicating with the Aimsun model, including:

- **int AAPILoad() {return 0;}** / the loading function;
- **int AAPIInit() {return 0;}** / the initialization function;
- **int AAPIManage() {return 0;}** / the main function called at initialization of a time step;
- **int AAPIPostManage() {return 0;}** / the main function called after completion of a time step;
- **int AAPIFinish() {return 0;}** / the finishing function;
- **int AAPIUnLoad() return 0;}** / the function for model uploading.

Aimsun loads the external application by calling the loading function, AAPILoad(), and basic parameters, including the name and path of external applications, are loaded using AAPIInit() function. After initialization, the main function of the external applications applied at the beginning of a time step is loaded by calling the AAPIManage() function, while the AAPIPostManage() function is called if the main function is to be applied after a simulation time step. The AAPIManage() function is used to input data for simulation, and the AAPIPostManage() function extracts, outputs, and saves analysis results. The AAPIFinish() function is used to finish the processing after all time steps, followed by the AAPIUnLoad which disconnects the simulation from the external application.

#### SDK module

**Fig 4:**
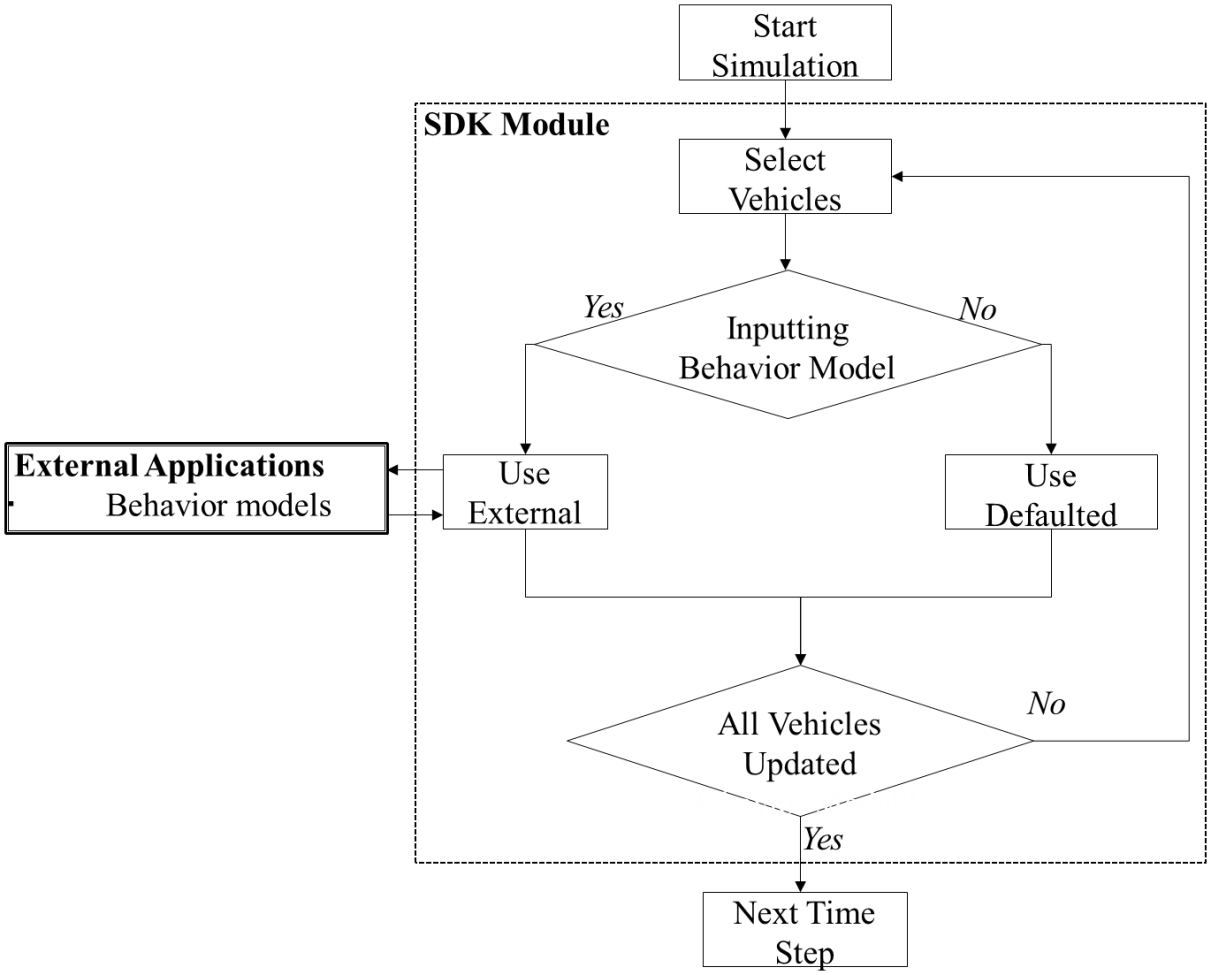
SDK module application flowchart.

The Aimsun Software Development Kit (SDK) module allows for the creation of user-defined applications (plugins) to override default behavioral models using the C++ programing platform [54]. Fig 4 presents how the SDK module loads external behavior model applications. In each time step, the simulation first selects vehicles for the behavior models. If external applications of behavior models are to be loaded, the SDK module will load the external applications and apply it to the behavior of the selected vehicles (otherwise, default internal models are used). The program checks if all the selected vehicles have been updated with the behavior model before moving on to the next time step.

### Simulating violations using Aimsun plugins

Violating behaviors were developed using the SDK module, shown in Fig 4, and loaded as presented in Fig 2. Users can select between default driving models or different violations types, which are assigned to random vehicles (1 in 500 vehicles were assigned violating behaviors). Plugins were developed for three moving violations; speeding, slow driving, and abrupt stopping. The plugin code for the violation models is provided in S1 Appendix. Further details are provided below.

#### Speed violation

Both slight and serious speeding violations were considered. Slight speeding was defined as vehicle speeds 25% over the speed limit, and serious speeding was 50% over the limit. Speeding vehicles maintain their speed unless inhibited by a leading vehicle, then change lanes or decelerate.

#### Slow driving violation

As with speeding, both slight and serious slow driving violations were investigated. Slight slow driving vehicles were assigned a speed equal to 50% of the speed limit. Serious slow driving vehicles speed was 25% of the limit. Slow driving vehicles always remain in the same lane.

#### Abrupt stop violation

A vehicle that stops abruptly increases crash risk by reducing reaction time and space for the following vehicle or by idling unexpectedly in the middle of the freeway. Randomly selected vehicles were to stop suddenly for five seconds or more, at a random deceleration rate between 3 and 7.5 m/s^2^.

### Monte Carlo method for rear-end crash probability

With simulation, accurate crash scenarios can be investigated without risking human lives. One objective of this paper is to investigate highway safety by estimating rear-end collision probability. A technique based on the Monte Carlo method is introduced to investigate rear-end collisions in a precise and microscopic way. Vehicle pairs were first classified according to four scenarios based on reaction and braking periods, according to the method proposed by Tsao and Hall [58]. The Monte Carlo method, a computerized mathematical technique that accounts for risk in quantitative analysis [59], is used to predict the collision probability of the vehicle pairs. This algorithm was developed and integrated using the API module, presented in Fig 2. The code for the Monte Carlo collision algorithm is provided in S2 Appendix.

#### Classification of rear-ending collisions

To address limitations of existing safety measures in traffic simulation and to bridge the gap between conflicts and collisions, this study utilizes the rear-ending collision model first proposed by Tsao and Hall [58]. The model considers reaction, braking, and the chance of colliding before vehicles come to a complete stop. Based on the status of the vehicle pairs at the moment of the crash, rear-end collisions can be classified according to four scenarios:

- **Scenario 1:** The following vehicle is not braking and the leading vehicle has not stopped.
- **Scenario 2:** The following vehicle is not braking and the leading vehicle has stopped.
- **Scenario 3:** The following vehicle is braking and the leading vehicle has not stopped.
- **Scenario 4:** The following vehicle is braking and the leading vehicle has stopped.

Assuming the SSD of the leading vehicle at time *t* is *D*_*l*_(*t*) and that of the following vehicle is *D*_*f*_(*t*), a collision occurs when:

$${\text{D}}_{1}(\text{t})+\text{L}={\text{D}}_{\text{f}}(\text{t})$$ (1)

where, *L* is the initial distance between the vehicles. The leading vehicle stops when *t* = *v*_*l*_/*a*_*l*_. Therefore, *t* > *v*_*l*_/*a*_*l*_ indicates that the leading vehicle has stopped, while *t* < *v*_*l*_/*a*_*l*_ indicates that the leading vehicle is still moving.

**Fig 5:**
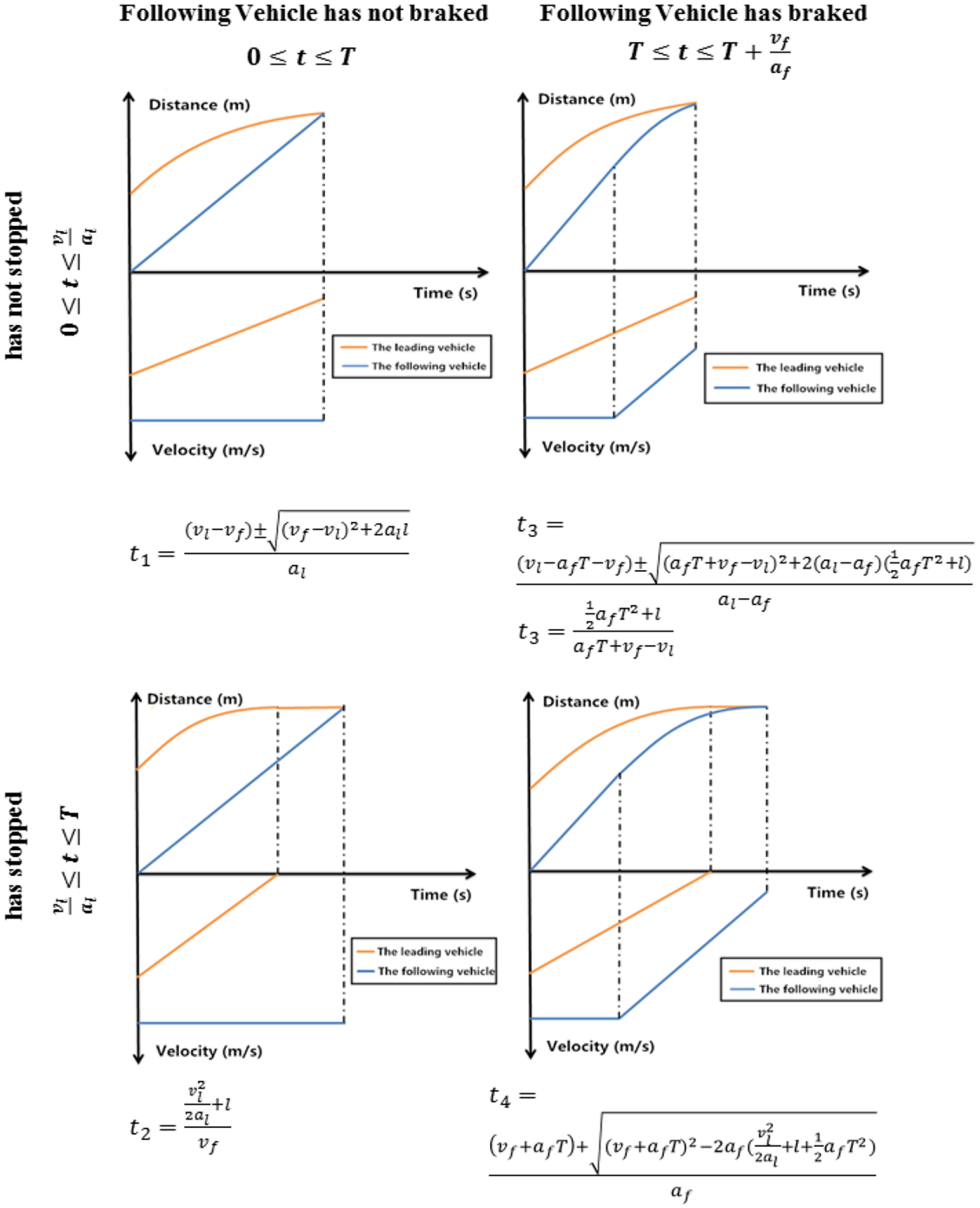
Descriptions of distance-time-velocity relationship in the four situations in the rear-end collision model.

Considering that actual braking reaction time (*T*) consists of both driver reaction time (*τ*) and time lag between activation (pressing the brake pedal) and the onset of deceleration (*θ*), *T* = *τ* + *θ*. For *t* < *T*, the following vehicle is not braking. If *t* > *T*, the following vehicle is braking. According to Eq (1) and the status of both the leading and following vehicles, the relationship between time, velocity, and acceleration of vehicle pairs, the specific crash scenario can be generated, as presented in Fig 5. Based on the scenario, the algorithm can calculate the type of collision and the time and location of the crash. The probability of rear-end collisions can then be predicted.

#### Collision probability of vehicle pairs

Gipps’ vehicle following model was designed for traffic simulation and model parameters were designed mainly to avoid collisions. In this study, a method was created for predicting collision probability between each vehicle pair as a necessity for investigating highway safety. For each vehicle, initial position and speed is extracted from the simulation. Behavioral parameters of deceleration rate and driver reaction time are random variables. The Monte Carlo method is a statistical simulation method based on the law of large numbers [60] that provides a good solution for investigating the probability of outcomes in a process involving random variables [59]. A large number of tests are conducted with different values for the random variables. Based on the outcomes of these tests, the probability of target outcome can be generated. For individual vehicle pairs, the probability of collision is the probability that the following vehicle will hit the front vehicle when the front vehicle conducts a braking maneuver. The deceleration rate of the leading vehicle is a random variable dependent on sociodemographic traits, as is the reaction time of the following vehicle. Avoiding a collision requires the driver of the following vehicle to decelerate at the maximum possible rate.

The Monte Carlo method selects random variables selected from a probability distribution. A normal distribution is best to describe deceleration rate of the leading vehicle (*a*_*l*_) [61]. *a*_*l*_ was assigned to be normally distributed with a mean of 5.2 m/s^2^ and a variance of 1 m/s^2^. Reaction time of the following vehicle (*T*) was assigned a log-normal distribution of LN(0.17, 0.44) as suggested by Wang [62]. Vehicle pairs were assigned one of the four crash scenarios and crash occurrence and time of the collision (*t*) were calculated based on the equations in Fig 5. The probability of collision, used as a measure of safety, was calculated as the number of tests resulting in collisions over the total number of tests. For each simulation step, the developed plugin targeted a certain pair of vehicles, extracted their trajectory, and obtained their positional and behavioral information. The plugin then applied the Monte Carlo method to calculate collision probability of the targeted vehicle pair.

### Predicting freeway crash risk

#### Road segment collision risk based on crash probability of individual vehicle pairs

Collision risk for road segments can be estimated based on the calculated crash probability of vehicle pairs. Segments are defined by evenly spaced virtual loops. In a given time period (simulation cycle), each segment contains several vehicle following cases. The time periods, which last several minutes, are based on the detection cycle of the virtual loops and cover several collision prediction simulation steps. Assuming that a simulation cycle contains m simulation steps for which there are n vehicles on the segment, the collision probability for every pair of vehicles can be written as an *m* × *n* matrix, presented in Table 1, where *r*_*ij*_ represents the collision probability of vehicle i in the simulation step j, determined using the Monte Carlo collision prediction model.

**Table 1:** Result matrix—vehicle rear-end collision probability on a certain segment in a certain simulation cycle.

	One Simulation Cycle				
	Step 1	Step 2	…	Step m-1	Step m
Vehicle 1	*r*_11_	*r*_12_	…	*r*_1,m-1_	*r*_1,m_
Vehicle 2	*r*_21_	*r*_22_		*r*_2,m-1_	*r*_2,m_
… …	*…*	*…*		*…*	*…*
Vehicle n-1	*r*_n-1,1_	*r*_n-1,2_		*r*_n-1,m-1_	*r*_n-1,m_
Vehicle n	*r*_n,1_	*r*_n,2_		*r*_n,m-1_	*r*_n,m_

Similar to [34], this study uses the 75^th^ percentile of collision probability to classify high crash risk. During a simulation cycle, for vehicle *j* in the *i*^*th*^ simulation step, the collision probability with the front vehicle is r_*ij*_, calculated using Monte Carlo method. Define the collision risk for the segment during the simulation step *i* (*r*_*i*_) as the 75^th^ percentile value

$$r_{i}=Q_{3}\left(r_{ij}\right),\left\{j=1,2,\ldots ,n\right\}$$ (2)

The collision risk on the road segment during this simulation cycle can be represented as the mean of the collision risk value of all simulation steps;

$$r=\frac{\sum_{i=1}^{m}r_{i}}{m}$$ (3)

#### Risk evaluation using Fuzzy C-Means Clustering Algorithm

Traffic risk analysis relies on long observation periods. Road traffic is dynamic and observations from several minutes do not represent the general level of safety for a given road segment. Therefore, experiments with a large number of simulation cycles are required to obtain sufficient data for safety analysis. This study employs a Fuzzy C-Means Clustering Algorithm to evaluate the safety condition of different freeway segments over the large number of repeated simulation cycles. A base case, containing no violations, was used to set a threshold between normal and high risk for the clustering procedure. Fuzzy Clustering is a common technique in data mining and machine learning for complex data sets [63]. The Fuzzy C-Mean Algorithm (FCM) is the most popular algorithm first proposed by Dunn [64] and improved by Bezdek [65]. The purpose of clustering is to find the centroid for different clusters and define which observations belong to which clusters. For a given data set {*x*_*i*_}, (*i* = 1,2,…,*N*), the FCM analysis steps are:

***Step 1*:** Select the number of clusters, *C*.
***Step 2*:** Select level of cluster fuzziness, *f>1*.
***Step 3*:** Initialize an *N* × *C* matrix (the fuzzy membership matrix, *U*) at random, with members *μ*_*ij*_ ∈ [0, 1]. For each *i*;
  $$\sum_{j=1}^{C}\mu_{ij}=1$$ (4)
***Step 4*:** Obtain the value function for clustering;
  $$\text{E}\left(\text{U},\text{V}\right)=\sum_{\text{i}=1}^{\text{C}}\sum_{\text{j}=1}^{\text{n}}{\left(\mu_{\text{ij}}\right)}^{\text{f}}{\left({\text{d}}_{\text{ij}}\right)}^{2}$$ (5)
  where, *U* is the fuzzy membership matrix, *V* = (*V*_1_,*V*_2_,…,*V*_*i*_,…,*V*_*C*_)^*T*^ is the matrix of the cluster centers, *V*_*i*_ is the vector of the cluster center for cluster *i*, *d*_*ij*_ is the Euclidean distance between data *j* and center of cluster *i*, expressed as *d*_*ij*_ = ||*x*_*j*_ − *V*_*i*_||.
***Step 5*:** Find the fuzzy membership matrix minimizing the value of *E*(*U*, *V*). Using the method of Lagrange multipliers, *E*(*U*, *V*) is minimized when:
  $$\mu_{\text{ij}}=\frac{1}{\sum_{\text{l=1}}^{\text{C}}{\left(\frac{{\text{d}}_{\text{ij}}}{{\text{d}}_{\text{lj}}}\right)}^{\frac{2}{\text{m}-1}}}\text{ l}=(1,2,\ldots ,\text{C})$$ (6)

Then, cluster centers are defined;

$${\text{V}}_{\text{i}}=\frac{\sum_{\text{j}=1}^{\text{N}}\left(\mu_{\text{ij}}\right){\text{x}}_{\text{j}}}{{\sum_{\text{j}=1}^{\text{N}}\left(\mu_{\text{ij}}\right)}^{\text{m}}}\text{ i},\text{j}=(1,2,\ldots ,\text{N})$$ (7)

Highway collision risks for different segments are analyzed by the FCM algorithm to determine the high-risk threshold. Different cluster numbers were tested, starting with 2 and incrementing until the largest cluster center value became stable. The largest cluster center value, *V*_*C*_, was selected as the high-risk threshold. Collision probability for each vehicle pair were simulated and input into Matlab. Collision risk on different segments during each simulation cycle were determined and clustered using the Fuzzy Logic Toolbox^™^ in Matlab. The largest cluster center value was extracted as the high-risk threshold. Sections with collision risk higher than this threshold were determined to be high risk, while sites with lower risk were considered normal.

## Simulation experiment

A simulation experiment was conducted to investigate the impact of vehicle moving violations on freeway safety. The experiment included various traffic conditions and volumes (1000, 1500, and 2000 veh/h) both with and without vehicle violations. Freeway crash risk high-risk thresholds were determined and used to identify segments with high collision risk under various traffic schemes.

### Introduction of simulation scenario & data output

**Fig 6:**
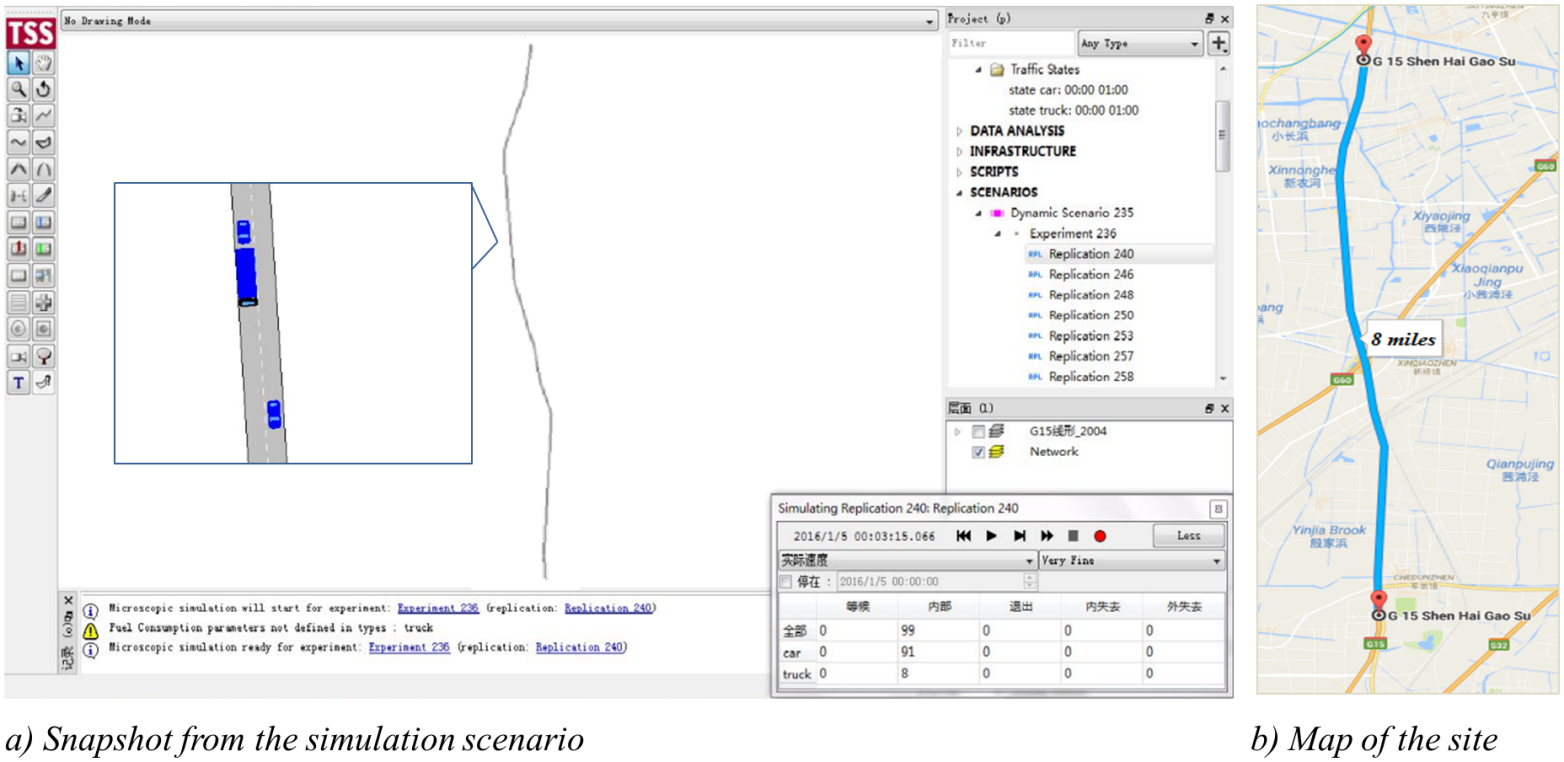
Aimsun interface of the simulated section.

The simulation scenario was based on the geometric design of a section of the G15 Shen Hai Expressway in Shanghai, presented in Fig 6. The section is 7 miles long with two lanes in each direction and without any on- or off-ramps to avoid the effect of merging. The design speed is 120 km/h with a posted speed limit of 100 km/h. Traffic data of speed, volume, and occupancy, were obtained using 15 virtual loop detectors at intervals of 0.5 miles, dividing the section into 14 segments. Traffic conditions included low volume (1000 veh/h), moderate volume (1500 veh/h) and high volume (2000 veh/h). Several variations of vehicle moving violation types were tested, including

1. No vehicle violation
2. Slight speeding violation (25% above limit)
3. Serious speeding violation (50% above limit)
4. Serious slow driving violation (25% of limit)
5. Slight slow driving violation (50% of limit)
6. Abrupt stop violation, idle for 5 s
7. Abrupt stop violation, idle for 10 s
8. Abrupt stop violation, idle for 20 s
9. Abrupt stop violation, idle for 40 s

Therefore, 27 different situations (3 traffic volumes with 9 violations) were tested. Each situation was simulated 50 times, each consisting of 24 simulation cycles of 2 minutes each. This results in 1200 collision risk measurements for each of the 14 segments, for all of the 27 traffic situations, totaling 453,600 observations (16,800 for each situation)

### Risk thresholds by Fuzzy C-Mean Algorithm

This study used data from the simulations with no vehicle violations to set the high-risk threshold. The low, moderate, and high volume simulations were considered, and collision risks for different segments were analyzed using the Fuzzy C-Mean Clustering Algorithm. Table 2 shows an example of collision risk data for each segment extracted from the simulation output. For each traffic condition, the risk threshold is calculated based on all 16,800 observations.

**Table 2:** Example of data for determining risk thresholds. Risk data from moderate traffic condition (Volume: 1500 veh/h).

No. of Simulation Cycle	Risk for Different Segment					
	*s_1_2*	*s_2_3*	*s_3_4*	*s_4_5*	*s_5_6*	*…*
1	0.2616	0.2779	0.2943	0.303	0.3102	…
2	0.2757	0.3312	0.3598	0.3809	0.3631	
3	0.3449	0.3331	0.3374	0.3306	0.3554	
4	0.3011	0.4048	0.4652	0.4845	0.4361	
5	0.3102	0.3208	0.3332	0.3499	0.4008	
6	0.291	0.2716	0.3085	0.3142	0.3835	
7	0.3698	0.3424	0.3753	0.376	0.3187	
8	0.3108	0.3306	0.3357	0.3887	0.4388	
9	0.3127	0.3376	0.3482	0.3337	0.3583	
10	0.2305	0.2513	0.2618	0.3074	0.3629	
11	0.2612	0.3161	0.3303	0.3394	0.3231	
12	0.3028	0.293	0.3044	0.2832	0.2862	
13	0.3283	0.3668	0.3783	0.3525	0.369	
14	0.2985	0.2985	0.3343	0.3275	0.3696	
15	0.3287	0.3338	0.3581	0.3896	0.3658	
16	0.3105	0.3456	0.3328	0.3499	0.3315	
…	……					

Note that: s_k_k+1 represents the segment between the kth and k+1th loop sensor

**Fig 7:**
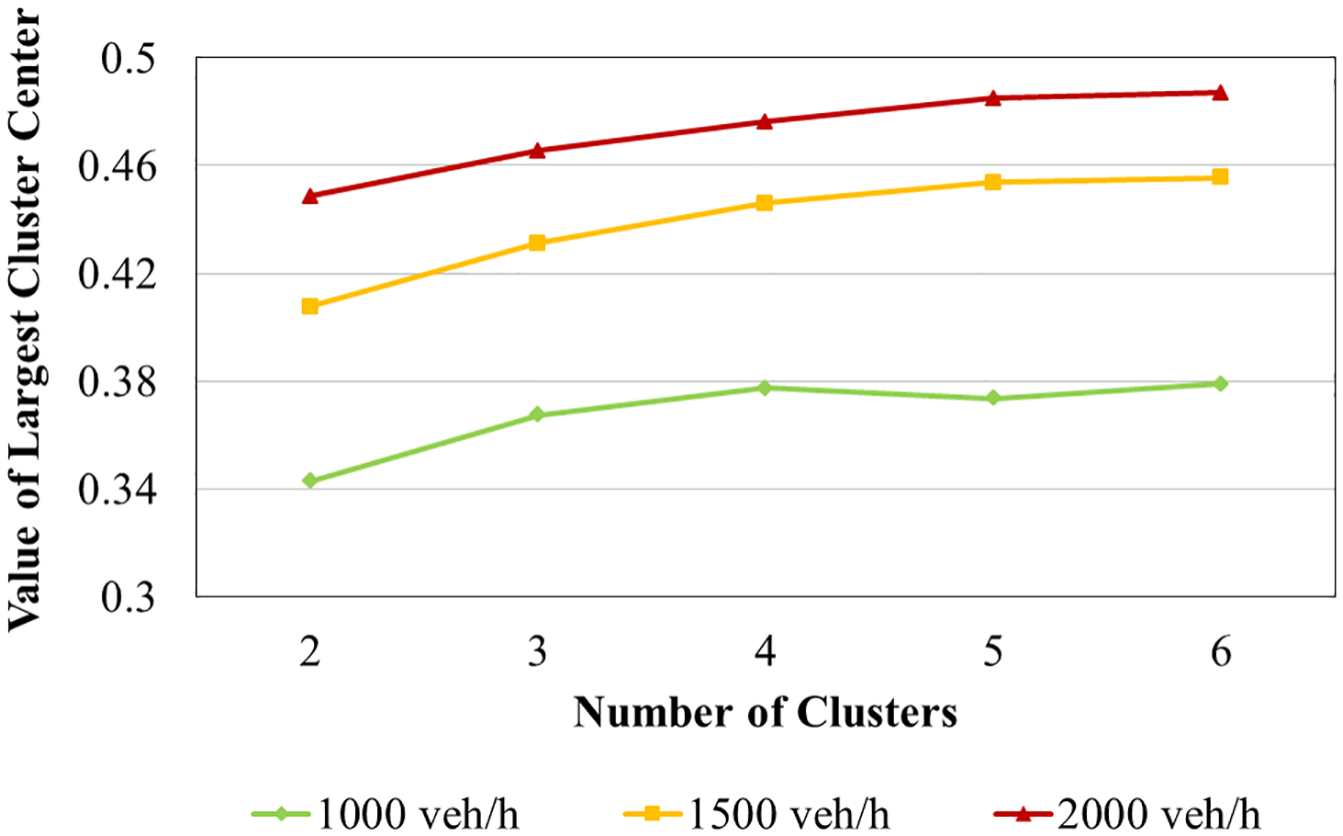
Largest cluster center value (*V*_*C*_) with different cluster number for different volume conditions.

Table 3 and Fig 7 present the values of the largest cluster centers for three different traffic flows and for different numbers of clusters. The largest value of the risk cluster center increased gradually before the number of clusters reached four, at which point the value stabilized. Accordingly, four clusters were used to group road section risks. Table 4 presents the values of the four cluster centers under three different flow conditions. The high-risk threshold is the largest cluster center value.

**Table 3:** Largest cluster center with different cluster number.

Traffic Volume	Cluster center value for number of clusters				
	*2*	*3*	*4*	*5*	*6*
1000 veh/h	0.3429	0.3673	0.3775	0.3735	0.3789
1500 veh/h	0.4076	0.4311	0.4460	0.4536	0.4555
2000 veh/h	0.4487	0.4654	0.4762	0.4849	0.4871

**Table 4:** Results of cluster centers.

Traffic Volume	Value of the cluster centers			
	*the 1*^*st*^	*the 2*^*nd*^	*the 3*^*rd*^	*the 4*^*th*^
**1000 veh/h**	0.2218	0.2805	0.3252	0.3775
**1500 veh/h**	0.2963	0.3428	0.3910	0.4460
**2000 veh/h**	0.3581	0.3981	0.4321	0.4762

### Results of impact vehicle violations types on freeway safety

For every traffic volume and violation input, the number of high-risk segments in each simulation cycle was determined over 50 simulations. The average number of high-risk segments was calculated and compared. Results of the 27 simulation situations are presented in Table 5.

**Table 5:** The number of high-risk status before and after adding vehicle violations for different traffic conditions.

Traffic Volume	Different Type of Traffic Violation								
	*No Violation*	*Speeding*		*slow driving*		*abrupt stop for 5 s*			
		*25%*	*50%*	*25%*	*50%*	*For 5 s*	*For 10 s*	*For 20 s*	*For 40 s*
**1000 veh/h**	9.34	9.78	9.38	9.52	19.56	9.98	14.58	14.4	14.82
**1500 veh/h**	45.46	48.38	49.56	57.68	65.86	136.02	155.28	155.12	156.96
**2000 veh/h**	33.52	37.48	41.34	61.72	107.4	104.96	105.52	106.46	106.17

**Fig 8:**
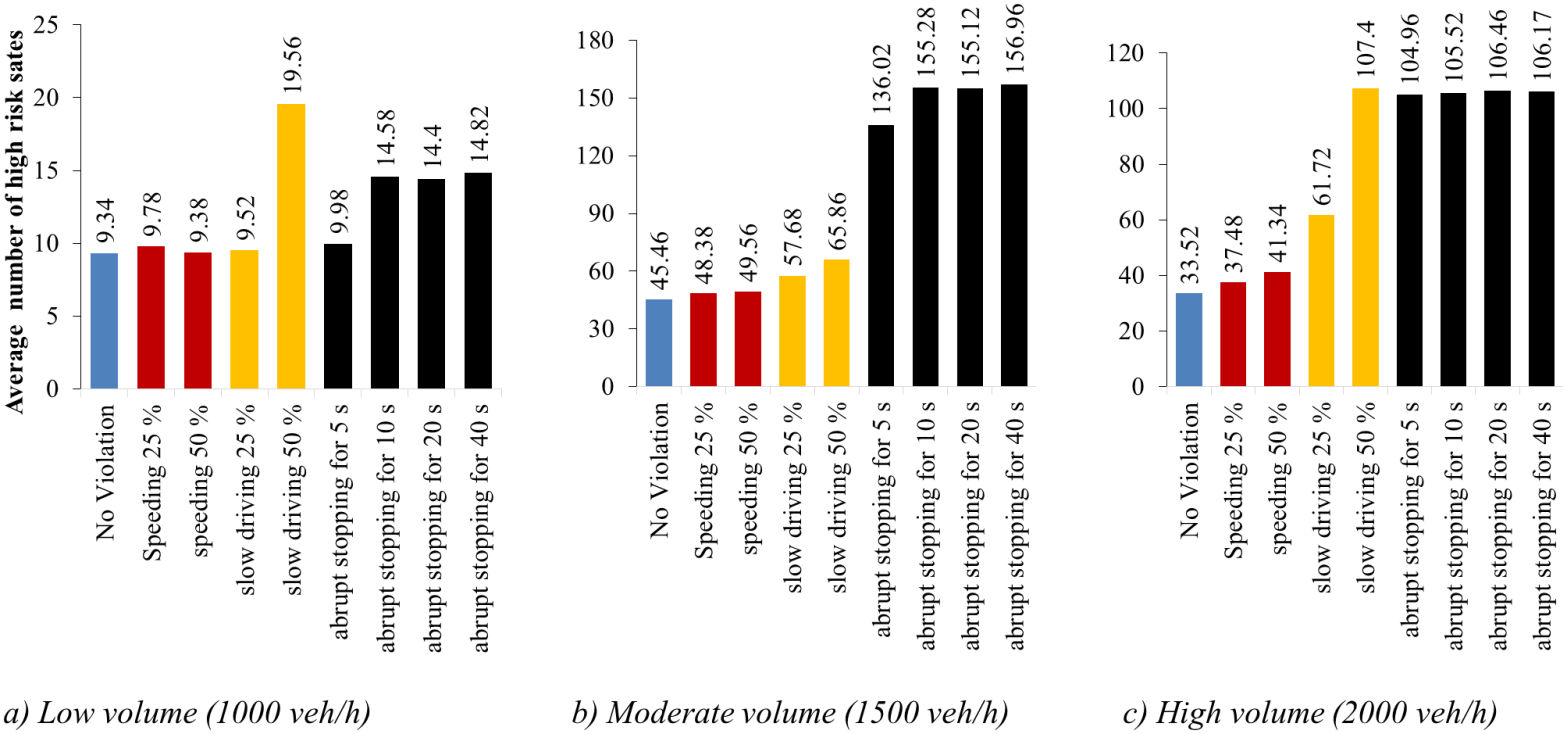
Comparisons of different types of violation.

Fig 8 shows the comparison of collision risk, represented by the average number of high-risk states, for different types of traffic violation. Several phenomena affect the impact of different violation types on freeway safety. Speeding vehicles increase risk mainly to surrounding vehicles. Speeding vehicles attempting to change lanes mainly affect the leading vehicle or vehicles in adjacent lanes. Slow driving and abrupt stopping vehicles affect both surrounding vehicles and overall traffic flow. Slow vehicles inhibit following vehicles and reduce the average speed, increasing traffic density and risk. Vehicles that stop abruptly have increased probability of crash with the following vehicle due to high deceleration and also increased traffic density and risk while idling.

Considering the impact of traffic volumes, in the low volume condition, presented in Fig 8a, slight speeding increased the number of high-risk segments by only 0.44 on, while serious speeding increased the risk by 0.04 compared to the base case of no violations. The magnitude of the effect of slight slow driving was similar. The greatest increase in risk was attributed to serious slow driving, with more than twice as many high-risk states observed. Abrupt stopping with 5 seconds of idling resulted in a minor increase in risk, while idling lengths greater than 5 seconds generally resulted in an increase in risk of approximately 55%.

Under moderate volumes, presented in Fig 8b, the effect of speeding and slow driving was similar compared to low volume conditions. Speeding increased the average high risk state counts by approximately 10%, slight slow driving had a 27% increase, and serious slow driving increased risk by 45%. Abrupt stopping led to the greatest increase in risk. The average number of high risk states increasing threefold or more for all idling lengths

In high volume conditions, presented in Fig 8c, speeding violations remained the least impactful, though the impact of serious speeding became more significant. Serious slow driving tripled the number of high risk states, while slight slow driving nearly doubled it. Again, abrupt stopping increased the average number of high risk states by a factor of three, regardless of idling length.

In general, collision risk was observed to increase with violation severity. Though the impact of speeding violations was significant, it was much smaller compared to other violations. Speeding violations are likely to affect surrounding vehicles, increasing risk for a small proportion of traffic, while other violations have more macroscopic impacts.

**Fig 9:**
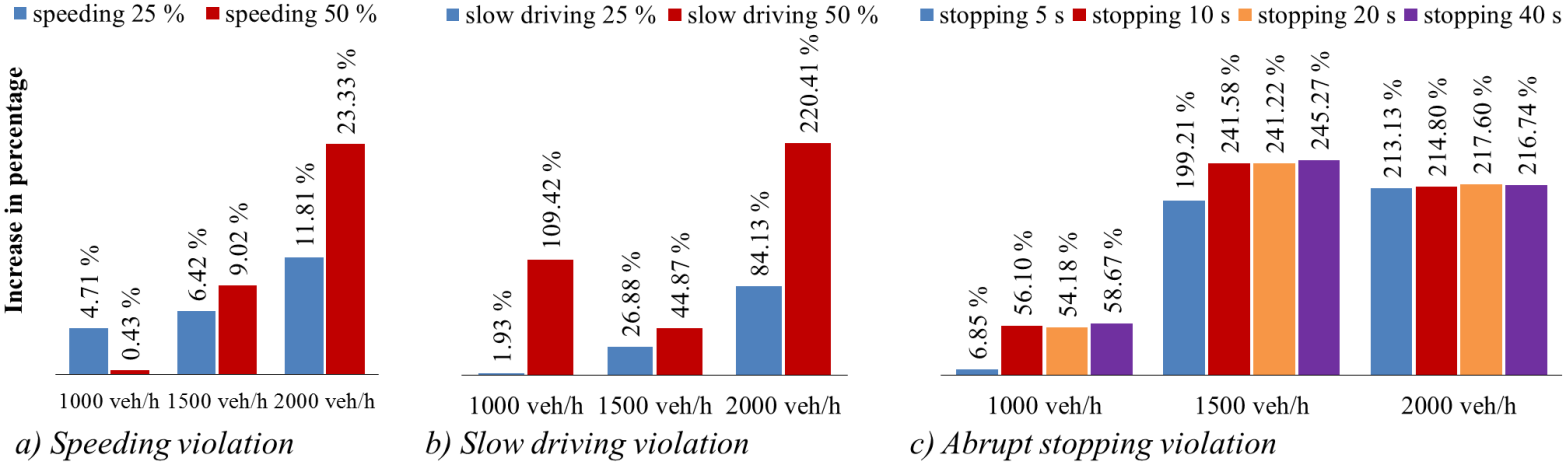
Influence of violation under different traffic volume.

The impact of violations on collision risk changes significantly with traffic volume. In general, slight driving violations in low volume have only a slight impact on safety. Risk increases both with violation severity and traffic volume. The percentage increase in observed high-risk states compared to the base case is used to represent the impact on collision risk in Fig 9. The impact of speeding violations increases with traffic volume, which is closely associated with traffic density and headways and determines freedom for accelerating and lane changing maneuvers. The impact of slow driving is most significant in high volume traffic. Though the percentage increase for serious slow diving violations under low traffic violation is significantly high, the absolute change is small. Abrupt stopping is less influential at low volumes than at moderate or high volumes.

### Spatio-temporal distribution of freeway risk

**Fig 10:**
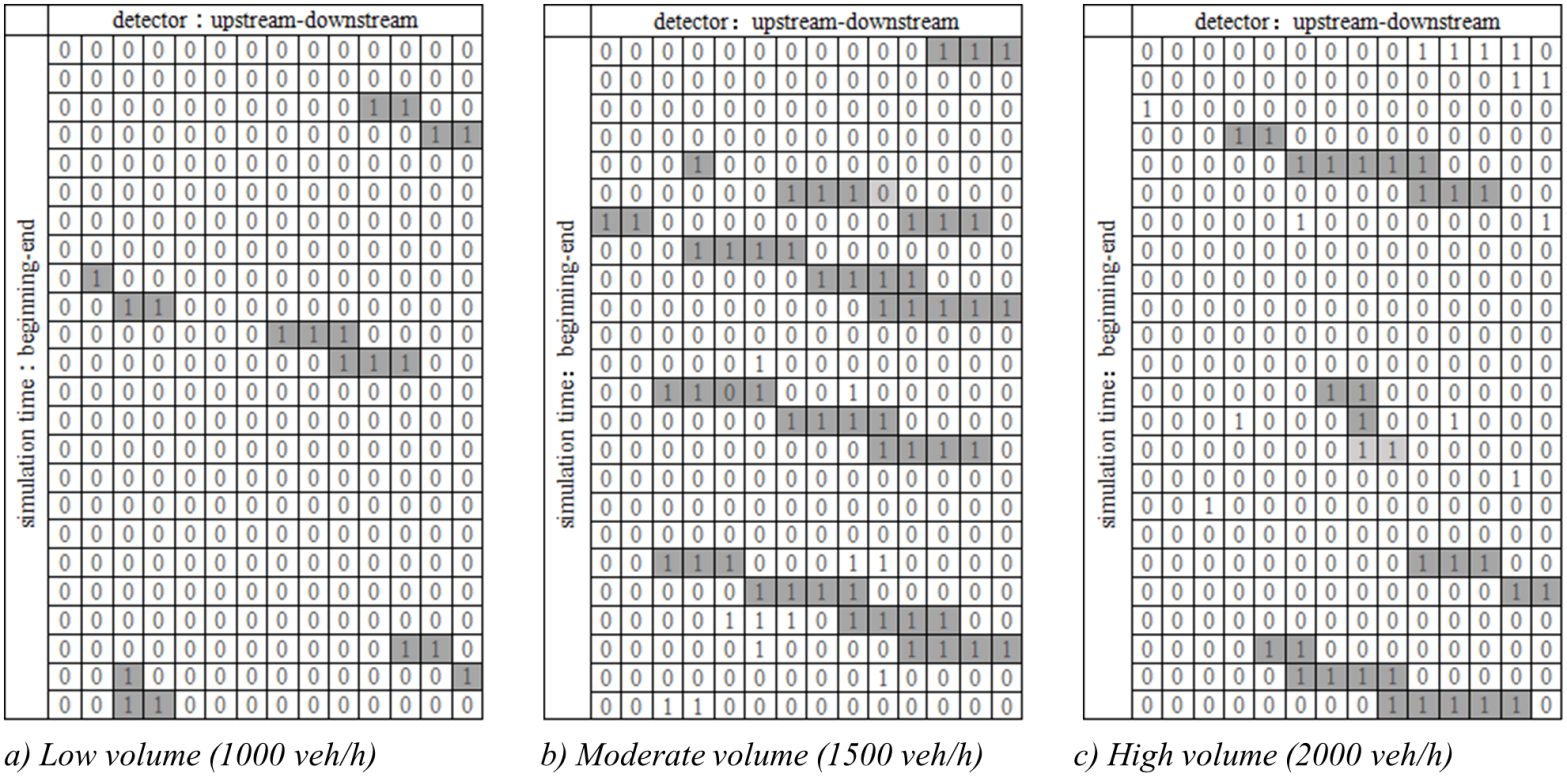
Space-time diagram under different traffic condition with no violations.

The risk states of road segments can be categorized, using 1 as the high-risk state and 0 as normal risk, and visualized using a space-time diagram. Here, time (simulation cycles) is set on the vertical axis from top to bottom, while distance down the highway (road segments) is horizontal from left to right. Conditions for the base case are presented in Fig 10 for the three traffic volumes. In the charts, high-risk states are observed to form continuous and semi-continuous diagonal bands as collision risk moves from upstream to downstream over time. Average simulated vehicles speed is around 100 km/h. Within a 2 minute simulation cycle, vehicles move approximately 3.3 km downstream, which covers 4 road segments (800 m each). This matches the observation in Fig 10.

**Fig 11:**
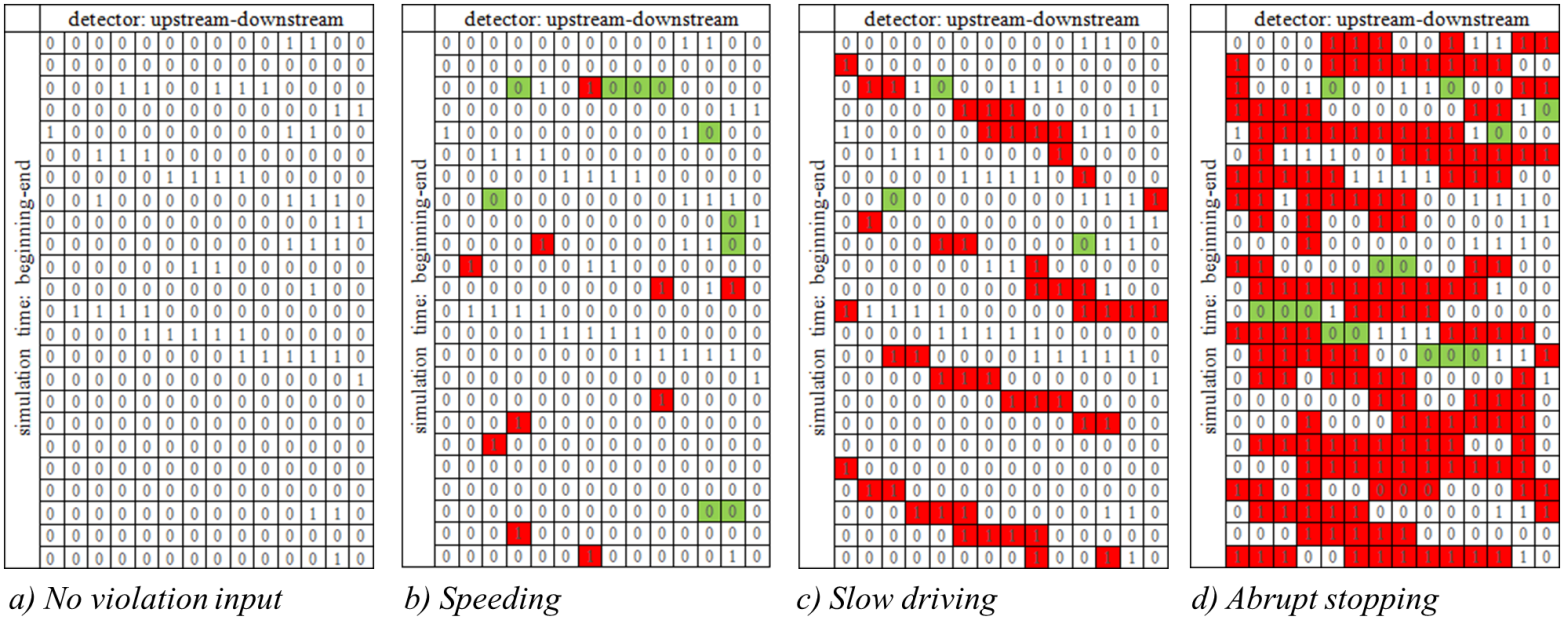
Space-time diagram of traffic flow risk for different violation types.

When violations are added, findings are similar to those above. Fig 11 shows the space-time diagrams for moderate traffic conditions with different traffic violation inputs. Fig 11a presents the base case and Fig 11b–11d provide results for the three violation types. In these figures a segment with increased risk compared to the base case is colored in red, while segments with decreased risk are colored green. Speeding violations created some new high risk states, while also eliminating some existing high risk states. The red boxes, which most likely represent the occurrences of violations, are scattered and isolated. This indicates that speeding violations mostly affect only surrounding vehicles. Results for slow driving violations clearly show the diagonal bands of high risk resulting from the violations, which implies a significant impact on passing traffic. The impact of abrupt stopping violations is comparatively greater than speeding and slow driving. This could be explained because high deceleration rates create high risks for surrounding vehicles, while idling continuously affects passing vehicles, increasing high risk cases not only diagonally but also vertically (over time while remaining stationary).

## Conclusions

The purpose of this paper was to develop a method for evaluating the effect of vehicle moving violations on freeway safety using a microsimulation program. Methodological and algorithm-based solutions for quantifying the impact of violations on rear-end collision risk are provided for the Aimsun simulation program. The Monte Carlo method is employed through the API and SDK modules, which are open to users for secondary development. Based on simulation output, this paper also presents a Fuzzy C-mean Clustering algorithm for identifying high risk collision states on freeway segments. A simulation experiment, which used the geometric design of a section on G15 Shen Hai Expressway in Shanghai, was conducted to illustrate these developed tools and methods. Several key conclusions are drawn from the above work.

- Aimsun, a widely used simulation platform, uses Gipps’ vehicle following and lane changing models to provide accurate microscopic traffic simulation. Algorithms for vehicle violations were built based on realistic maneuvers, simulation outputs appeared reasonable, and safety results are explained by traffic flow theory.
- The impact of different violations varies with traffic volume. The impact of speeding is not as significant as other violations, given that speeding vehicles mainly affect surrounding vehicles rather than affecting traffic in general. Slow driving and abrupt stopping continuously block passing vehicles, increasing crash risk especially when other vehicles attempt to overtake.
- As volume increases, density increases and headways decrease, and less space is available for lane changing or adaptive maneuvers. This explains why the impact of violations is closely related to increases in volume.
- Space-time diagrams of collision risk state demonstrate how collision risks change over time and space. Crash risk normally moves downstream over time. Risk from speeding violations tend to be isolated, and do not move diagonally in the diagram. Slow driving and abrupt stopping have a continuous and higher impact on traffic.

As key contributions, plugins were created for simulating traffic violations, one of the most important factors leading to freeway crashes which are difficult to investigate in real road environments. This study improves computer-based technologies for simulating and examining road user behavior, especially on freeways where severe crashes are more likely. Using simulation and the violation plugins, rich vehicle trajectory data can be continuously extracted for detailed investigation of the impact of violations in different traffic environments, which has previously been conducted through field observations or video-based tracking techniques at single sites [40] [66]. The paper provides a method for investigating the impact of microscopic traffic behaviors on macroscopic safety, which has rarely been explored, and provides a detailed and novel framework for investigating freeway risks based on the trajectory data extracted from the simulation. Though three types of violations were considered, the methods could easily be extended to other behaviors. The Monte Carlo method provides a promising solution for predicting crash risk between vehicle pairs with consideration for different random variables. In practice, these methods can be applied for purposes including hazard prediction and monitoring, evaluation of road design, and hotspot identification.

In the future, the entire methodology should be validated and further calibrated using historical crash data. The simulation algorithms could be improved to model violations and consider additional factors with the development of simulation technologies. New technologies, such as LiDAR sensors, may enable the study of vehicle violations in real road environments which may assist the development of violation models. Though reliable, Gipps’ vehicle following and lane changing models could be further calibrated to better describe driver behavior when violations are involved, or substituted with better models. The method of using space-time diagrams can be further explored, and the safety evaluation measures can be further validated. Lastly, though rear-end collisions are the most common type of crashes on freeway conditions, other collisions cannot be ignored, and future work must consider other collision types.

## Supporting information

S1 Appendix Programing for violation plugins. (DOCX)

S2 Appendix Programing for Monte Carlo rear-end collision simulation. (DOCX)

S1 Data Aimsun simulation inputs and calibrations. (DOCX)

S2 Data Sample of simulation output. (XLSX)
