# Supplementary material for: The impact of vehicle moving violations and freeway traffic flow on crash risk: An application of plugin development for microsimulation
Source: PLoS One. 2017 Sep 8;12(9):e0184564. doi: 10.1371/journal.pone.0184564 (PMC5590972; doi:10.1371/journal.pone.0184564)
Supplement: S1 Appendix — (DOCX) [file pone.0184564.s001.docx]

# Appendix 1 Programing for Violation Plugins

## 1-I Speeding Violation Plugin

void mybehavioralModel::updateVehicle( A2SimVehicle *vehicle_overspeed)

{

if ((!vehicle_overspeed->isFictitious()) && (vehicle_overspeed->getId() % 500 == 0))

//One speeding vehicle among every 500 vehicles (with a rate of 0.2%)

{

int vehdownid(A2SimVehicle* target_vehicle,int target_lane);

int vehupid(A2SimVehicle* target_vehicle,int target_lane);

const A2SimVehicle *leader = vehicle_overspeed->getLeader();

double t_stepcycle = AKIGetSimulationStepTime();

int id_f = vehicle_overspeed->getId();

double v_f = vehicle_overspeed->getSpeed(0);

double v_f_ideal = 150/3.6; //Defining the speed of speeding violation (25% and 50% over the speed limit)

double a_f_acc_max = AKIVehGetStaticInf(id_f).maxAcceleration;

double a_f_dec_max = AKIVehGetStaticInf(id_f).maxDeceleration;

double pos_f = vehicle_overspeed->getPosition(0);

double t_r_f = 0;

double v_l = leader->getSpeed(0);

double a_l_dec_est = AKIVehGetStaticInf(leader->getId()).maxDeceleration;

double length_l = leader->getLength();

double pos_l = leader->getPosition(0);

int current_lane = vehicle_overspeed->getIdCurrentLane();

InfVeh overspeed_infveh = AKIVehGetInf(id_f);

int overspeed_sec_id = overspeed_infveh.idSection;

A2KSectionInf overspeed_secinf = AKIInfNetGetSectionANGInf(overspeed_sec_id);

int overspeed_lane_nb = overspeed_secinf.nbCentralLanes;

// Speed calculation using Gipps vehicle-following model

double v_free = v_f + 2.5* a_f_acc_max * t_stepcycle * (1- v_f/v_f_ideal) * sqrt(0.025+ v_f/v_f_ideal);

double v_constrain = a_f_dec_max * (t_stepcycle/2 + t_r_f) + sqrt( pow(a_f_dec_max * (t_stepcycle/2 + t_r_f),2) - a_f_dec_max *(2*(pos_l - length_l - pos_f) - v_f * t_stepcycle - pow(v_l,2)/a_l_dec_est));

double v_gipps;

if (v_free>=v_constrain)

v_gipps = v_constrain;

else

v_gipps = v_free;

if ((!leader->isFictitious()))

{

if (v_f>=v_gipps)

{

int left_lane = 1;

int right_lane = -1;

const A2SimVehicle *vehUp = NULL;

const A2SimVehicle *vehDown = NULL;

int vehdown_id;

InfVeh vehicle_down;

double distance_leader;

double distance_vehdown;

if (current_lane == 1) // 1 indicates the rightmost lane

{

vehdown_id = vehdownid(vehicle_overspeed,left_lane);

if (vehdown_id>0)

{

vehicle_down = AKIVehGetInf(vehdown_id);

distance_leader = pos_l - pos_f - length_l;

distance_vehdown = vehicle_down.CurrentPos - pos_f - AKIVehGetStaticInf(vehdown_id).length;

vehicle_overspeed->getUpDown(vehUp,vehDown,left_lane,0);

if ((distance_vehdown>distance_leader) && vehicle_overspeed->isLaneChangingPossible(left_lane))

vehicle_overspeed->applyLaneChanging(vehDown,left_lane,pos_f + v_f * t_stepcycle,v_f);

else

vehicle_overspeed->setNewPosition(pos_f + v_f * t_stepcycle,v_gipps);

}

else

vehicle_overspeed->applyLaneChanging(vehDown,left_lane,pos_f + v_f * t_stepcycle,v_f);

}

else if (current_lane == overspeed_lane_nb) // n is the number of the lanes, here, indicates the leftmost lane

{

vehdown_id = vehdownid(vehicle_overspeed,right_lane);

if (vehdown_id > 0)

{

vehicle_down = AKIVehGetInf(vehdown_id);

distance_leader = pos_l - pos_f - length_l;

distance_vehdown = vehicle_down.CurrentPos - AKIVehGetStaticInf(vehdown_id).length - pos_f;

vehicle_overspeed->getUpDown(vehUp,vehDown,right_lane,0);

if ((distance_vehdown>distance_leader) && vehicle_overspeed->isLaneChangingPossible(right_lane))

vehicle_overspeed->applyLaneChanging(vehDown,right_lane,pos_f + v_f * t_stepcycle,v_f);

else

vehicle_overspeed->setNewPosition(pos_f + v_f * t_stepcycle,v_gipps);

}

else

vehicle_overspeed->applyLaneChanging(vehDown,right_lane,pos_f + v_f * t_stepcycle,v_f);

}

else

{

vehdown_id = vehdownid(vehicle_overspeed,left_lane);

if (vehdown_id>0)

{

vehicle_down = AKIVehGetInf(vehdown_id);

distance_leader = pos_l - pos_f - length_l;

distance_vehdown = vehicle_down.CurrentPos - pos_f - AKIVehGetStaticInf(vehdown_id).length;

vehicle_overspeed->getUpDown(vehUp,vehDown,left_lane,0);

if ((distance_vehdown>distance_leader) && vehicle_overspeed->isLaneChangingPossible(left_lane))

vehicle_overspeed->applyLaneChanging(vehDown,left_lane,pos_f + v_f * t_stepcycle,v_f);

else

{

vehicle_down = AKIVehGetInf(vehdown_id);

distance_leader = pos_l - pos_f - length_l;

distance_vehdown = vehicle_down.CurrentPos - pos_f - AKIVehGetStaticInf(vehdown_id).length;

vehicle_overspeed->getUpDown(vehUp,vehDown,right_lane,0);

if ((distance_vehdown>distance_leader) && vehicle_overspeed->isLaneChangingPossible(right_lane))

vehicle_overspeed->applyLaneChanging(vehDown,right_lane,pos_f + v_f * t_stepcycle,v_f);

else

vehicle_overspeed->setNewPosition(pos_f + v_f * t_stepcycle,v_gipps);

}

}

else

vehicle_overspeed->applyLaneChanging(vehDown,left_lane,pos_f + v_f * t_stepcycle,v_f);

}

}

else

vehicle_overspeed->setNewPosition(pos_f + v_f * t_stepcycle,v_free);

}

else

vehicle_overspeed->setNewPosition(pos_f + v_f * t_stepcycle,v_free);

}

}

int vehdownid(A2SimVehicle* target_vehicle,int target_lane)

{

int id = -1;

int sect_nb = AKIInfNetNbSectionsANG();

InfVeh infveh;

InfVeh tempveh;

for(int i=0;i<sect_nb; i++)

{

int sect_id = AKIInfNetGetSectionANGId(i);

int veh_nb = AKIVehStateGetNbVehiclesSection(sect_id, false);

for(int j=0; j<veh_nb; j++)

{

infveh = AKIVehStateGetVehicleInfSection(sect_id,j);

if((id==-1) && (infveh.numberLane==target_vehicle->getIdCurrentLane()+target_lane) && (infveh.CurrentPos > target_vehicle->getPosition(0)))

{

id = infveh.idVeh;

tempveh = infveh;

}

else if ((infveh.numberLane==target_vehicle->getIdCurrentLane()+target_lane) && (infveh.CurrentPos > target_vehicle->getPosition(0)) && (infveh.CurrentPos <tempveh.CurrentPos))

{

id = infveh.idVeh;

tempveh = infveh;

}

}

}

return id; // id is -1, or the id of the front-vehicle

}

## 1- II Slow Driving Violation Plugin

void mybehavioralModel::updateVehicle( A2SimVehicle *vehicle_lowspeed)

{

if ((!vehicle_lowspeed->isFictitious()) && (vehicle_lowspeed->getId() % 500 == 0))

//One speeding vehicle among every 500 vehicles (with a rate of 0.2%)

{

int vehdownid(A2SimVehicle* target_vehicle,int target_lane);

int vehupid(A2SimVehicle* target_vehicle,int target_lane);

const A2SimVehicle *leader = vehicle_lowspeed->getLeader();

double t_stepcycle = AKIGetSimulationStepTime();

int id_f = vehicle_lowspeed->getId();

double v_f = vehicle_lowspeed->getSpeed(0);

double v_f_ideal = 60/3.6; //Defining the speed of slow driving violation (25% and 50% of the speed limit)

double a_f_acc_max = AKIVehGetStaticInf(id_f).maxAcceleration;

double a_f_dec_max = AKIVehGetStaticInf(id_f).maxDeceleration;

double pos_f = vehicle_lowspeed->getPosition(0);

double t_r_f = 0;

double v_l = leader->getSpeed(0);

double a_l_dec_est = AKIVehGetStaticInf(leader->getId()).maxDeceleration;

double length_l = leader->getLength();

double pos_l = leader->getPosition(0);

int current_lane = vehicle_lowspeed->getIdCurrentLane();

InfVeh overspeed_infveh = AKIVehGetInf(id_f);

int overspeed_sec_id = overspeed_infveh.idSection;

A2KSectionInf overspeed_secinf = AKIInfNetGetSectionANGInf(overspeed_sec_id);

int overspeed_lane_nb = overspeed_secinf.nbCentralLanes;

//Speed calculation using Gipps vehicle-following model

double v_free = v_f + 2.5* a_f_acc_max * t_stepcycle * (1- v_f/v_f_ideal) * sqrt(0.025+ v_f/v_f_ideal);

double v_constrain = a_f_dec_max * (t_stepcycle/2 + t_r_f) + sqrt( pow(a_f_dec_max * (t_stepcycle/2 + t_r_f),2) - a_f_dec_max *(2*(pos_l - length_l - pos_f) - v_f * t_stepcycle - pow(v_l,2)/a_l_dec_est));

double v_gipps;

if (v_free>=v_constrain)

v_gipps = v_constrain;

else

v_gipps = v_free;

if ((!leader->isFictitious()))

{

if (v_f>=v_gipps)

{

int left_lane = 1;

int right_lane = -1;

if (vehicle_lowspeed->isLaneChangingPossible(left_lane))

vehicle_lowspeed->applyLaneChanging(vehDown,left_lane,pos_f + v_f * t_stepcycle,v_f);

else

vehicle_lowspeed->applyLaneChanging(vehDown,right_lane,pos_f + v_f * t_stepcycle,v_f);

}

else

vehicle_lowspeed->setNewPosition(pos_f + v_f * t_stepcycle,v_free);

}

}

## 1- III Abrupt Stopping Violation

void mybehavioralModel::updateVehicle( A2SimVehicle *vehicle_stop)

{

if ((!vehicle_stop->isFictitious())&& (vehicle_stop->getId() % 500 ==400))

//One speeding vehicle among every 500 vehicles (with a rate of 0.2%)

{

double t_stepcycle = AKIGetSimulationStepTime();

double global_time=AKIGetIniSimTime();

int id_f = vehicle_stop->getId();

InfVeh stop_infveh = AKIVehGetInf(id_f);

StaticInfVeh stop_sinfveh=AKIVehGetStaticInf(id_f);

double Enter_t=stop_infveh.SectionEntranceT;

double v_now = vehicle_stop->getSpeed(0);

double break_time = stop_sinfveh.maxDesiredSpeed;

double normal_dec=stop_sinfveh.normalDeceleration;

double pos_now = vehicle_stop->getPosition(0);

double v_next;

double stay_time = 5; // Setting the time duration for stopping (5, 10, 20 and 40 sec were used)

if ((global_time > Enter_t + 300) && (global_time < Enter_t + 300 + stay_time + break_time))

{

double v_dec = v_now - fabs(normal_dec)*t_stepcycle;

if (v_dec > 0)

v_next = v_dec;

else

v_next = 0;

vehicle_stop->setNewPosition(pos_now + v_now * t_stepcycle,v_next);

}

}

}
