# Supplementary material for: The impact of vehicle moving violations and freeway traffic flow on crash risk: An application of plugin development for microsimulation
Source: PLoS One. 2017 Sep 8;12(9):e0184564. doi: 10.1371/journal.pone.0184564 (PMC5590972; doi:10.1371/journal.pone.0184564)
Supplement: S2 Appendix — (DOCX) [file pone.0184564.s002.docx]

# Appendix 2 Programing for Monte Carlo Rear-end Collision Simulation

file_dir='d:\datafile ';

datus=importdata(fullfile(file_dir,'vehicle.txt'));

result=zeros(length(datus),5);

parfor i=1:length(datus)

L0=datus(i,7)-datus(i,3)-datus(i,10);

vl=datus(i,8);

vf=datus(i,4);

count=0;

times=1000; % setting the time of repetitions in Monte Carlo method

lv_type=datus(i,9);

fv_type=datus(i,5);

% calculate the head way if front vehicle exist; otherwise, collision possibility is 0

if (datus(i,6)==-1)

result(i,:)=[datus(i,1),datus(i,2),datus(i,3),datus(i,11),0]; % output data

continue

end

% calculate the collision possibility as the number of collisions over total repetitions

for j=1:times

al_car = -normrnd(5.2,1);

al_truck = -normrnd(4.5,1);

al=[al_car,al_truck];

if lv_type==1;

if and(al_car<=-3,al_car>=-7.5)

else

times=times+1;

continue

end

else

if and(al_truck<=-2.5,al_truck>=-6.5)

else

times=times+1;

continue

end

end

af=-normrnd(6.5,1);

tr=lognrnd(0.17,0.44);

tb=[0.175,0.65];

T=tr+tb(fv_type);

%-----------probability calculation based on the four types of collision----------

% 1. (leading vehicle has not stopped & following vehicle has not start to brake)

p1=roots([0.5*al(lv_type) vl-vf L0]);

if isreal(p1)==1

if p1(1)<T && p1(1)<(-vl/al(lv_type)) && p1(1)>0

count=count+1;

continue

else if p1(2)<T && p1(2)<(-vl/al(lv_type)) && p1(2)>0

count=count+1;

continue

end

end

end

% 2. (leading vehicle has stopped & following vehicle has not start to brake)

p2=-vl^2/(2*al(lv_type)*vf)+L0/vf;

if p2<T && p2>=(-vl/al(lv_type))

count=count+1;

continue

end

% 3. (leading vehicle has not stopped & following vehicle has started to brake)

p3=roots([0.5*(af-al(lv_type)) vf-af*T-vl 0.5*af*T^2-L0]);

if af~=al(lv_type)

if isreal(p3)==1

if p3(1)<=(-vf/af+T) && p3(1)<(-vl/al(lv_type)) && p3(1)>=T

count=count+1;

continue

else if p3(2)<=(-vf/af+T) && p3(2)<(-vl/al(lv_type)) && p3(2)>=T

count=count+1;

continue

end

end

end

else if p3(1)<=(-vf/af+T) && p3(1)<(-vl/al(lv_type)) && p3(1)>=T

count=count+1;

continue

end

end

% 4. (leading vehicle has stopped & following vehicle has started to brake)

p4=roots([0.5*af vf-af*T 0.5*af*T^2+vl^2/(2*al(lv_type))-L0]);

if isreal(p4)==1

if p4(1)<=(-vf/af+T) && p4(1)>=(-vl/al(lv_type)) && p4(1)>=T

count=count+1;

continue

else if p4(2)<=(-vf/af+T) && p4(2)>=(-vl/al(lv_type)) && p4(2)>=T

count=count+1;

continue

end

end

end

end

possibility=count/times;

result(i,:)=[datus(i,1),datus(i,2),datus(i,3),datus(i,11),possibility];%输出结果（2/2）

end
