# Supplementary material for: The impact of vehicle moving violations and freeway traffic flow on crash risk: An application of plugin development for microsimulation
Source: PLoS One. 2017 Sep 8;12(9):e0184564. doi: 10.1371/journal.pone.0184564 (PMC5590972; doi:10.1371/journal.pone.0184564)

**Supporting Information – Aimsun Simulation Inputs and Calibrations**

**1. Parameters for Road Scenario**

The first step in the simulation is to set up the road scenario. As included in the manuscript, the simulation scenario was based on the geometric design of a section of the G15 Shen Hai Expressway in Shanghai. With the map for the studied section, the simulation scenario was drawn in the Aimsum Program. Parameters, including the maximum speed, the capacity, and the volume delay function (VDF) were set as the basic input for the road scenario. Detailed settings for the parameters are included in Figure 1.


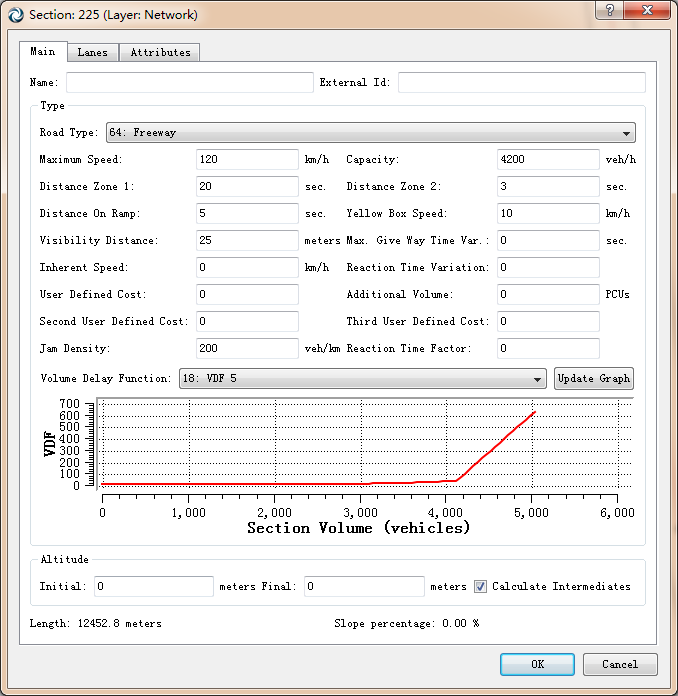


**Figure 1 Simulation Input – Parameters for Road Scenario**

**2. Parameters for Vehicles**

The second step in the simulation is to set up the parameters for vehicles. Parameters for vehicles include the length, width, maximum desired speed, maximum acceleration rate, maximum deceleration rate and etc. for the vehicles. The input information of these parameters contains the mean, the deviation, and the minimum and maximum values. Detailed settings for the parameters are presented in Figure 2.


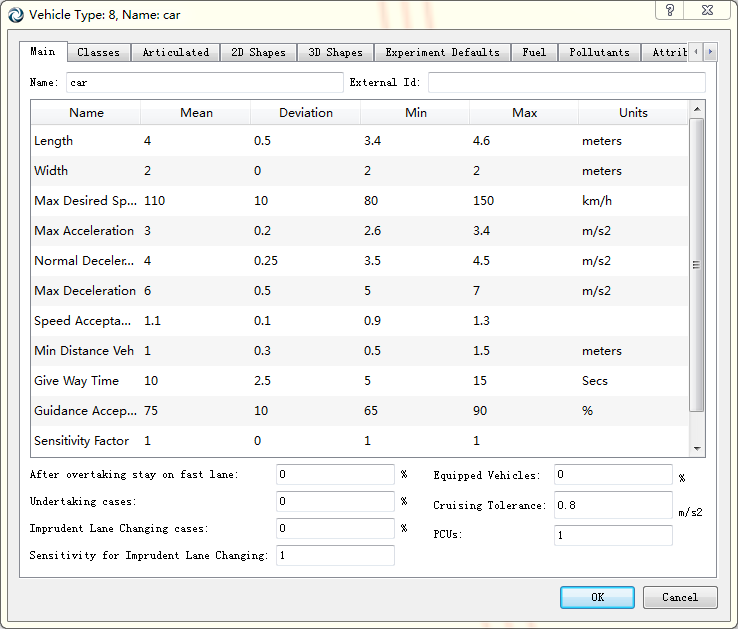


**Figure 2 Simulation Input – Parameters for Vehicle**

**3. Parameters for Driving Behavior**

The third step in the simulation is to set up the parameters for driving behavior. Driving behavior models in the Aimsum Program mainly includes the car following model and the lane changing model. Detailed of the settings about the related parameters for driving behavior are included in Figure 3. To study vehicle violations, violation plugins introduced in the manuscript can be loaded by selecting the “Activate External Behavior Model” option.


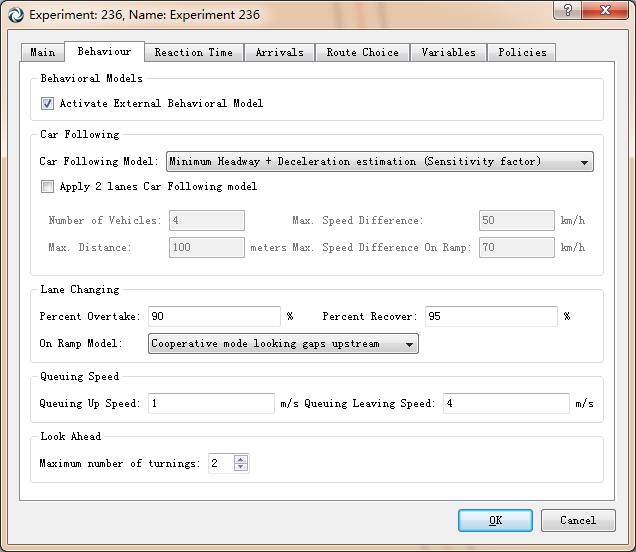


**Figure 3 Simulation Input – Parameters for Driving Behavior**

**4. Vehicle Tracking Data Collection**

The three steps above are about the main settings for the simulation. As included in the manuscript, the Aimsum simulation program uses time steps with a 0.1-second time length, which means that the program updated the status of all the vehicles every 0.1 second. Based on each time step, the study recorded the status of all the simulated vehicles every 0.1 second with which we are able to extract the trajectory of each vehicle. Table 1 gives a sample of status information recorded. Information for each vehicle includes the simulation time step where the vehicle status is recorded, the ID of the vehicle, the lane where it is in, speed at which it is driving at, the length of its leading vehicle, and the collision possibility with the leading vehicle as explained in the manuscript.

**Table 1 Vehicle Tracking Data Collection**


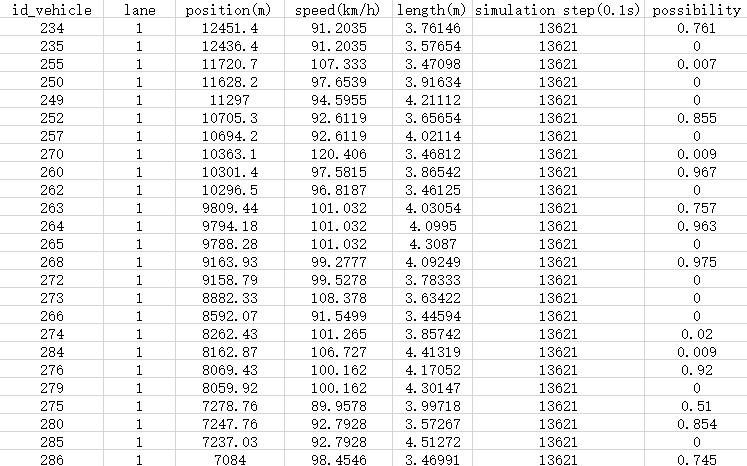

Supplement: S1 Data — (DOCX) [file pone.0184564.s003.docx]
